# Reckless Generosity, Parkinson's Disease and Dopamine: A Case Series and Literature Review

**DOI:** 10.1002/mdc3.13156

**Published:** 2021-02-24

**Authors:** Deborah Amstutz, Joan Philipp Michelis, Ines Debove, Marie Elise Maradan‐Gachet, Martin Lenard Lachenmayer, Julia Muellner, Kyrill Schwegler, Paul Krack

**Affiliations:** ^1^ Department of Neurology University Hospital Bern, University of Bern Freiburgstrasse 18 3010 Bern Switzerland; ^2^ Graduate School for Health Sciences University of Bern Mittelstrasse 43 3012 Bern Switzerland

**Keywords:** Parkinson's disease, dopamine, generosity, impulse control disorders, behavioral addiction, altruistic behavior

## Abstract

**Background:**

Impulse control disorders (ICDs) are a frequent side effect of dopamine replacement therapy (DRT) in Parkinson's disease (PD). Reckless generosity might expand the spectrum of known ICDs.

**Cases:**

Over 18 months, we encountered three PD patients exhibiting reckless generosity under DRT, leading to disastrous financial and social consequences.

**Literature Review:**

Except for another case series describing reckless generosity in three PD patients, only one study has examined generosity in PD patients; with findings suggesting that PD patients with ICDs are less sensitive to the aversive aspects of the lack of reciprocation in social settings. Studies with healthy individuals suggest that increased availability of dopamine might reduce social discounting and promote egalitarian behavior, and thereby increase generous behavior towards strangers. Genetic studies show that polymorphisms in dopamine D4 receptors influence generous behavior.

**Conclusions:**

Reckless generosity in PD patients with DRT might be underreported and should therefore be carefully be screened for by clinicians. A potential mechanism underlying this ICD‐related behavior might be a sensitization of the mesolimbic and mesocortical dopaminergic system, leading to reduced social discounting and maladaptive reward‐learning. Further research is needed to investigate the prevalence and underlying mechanisms of reckless generosity in PD patients.

Impulse control disorders (ICDs) are a well‐known side effect of dopamine replacement therapy (DRT) in Parkinson's disease (PD) patients; including pathological gambling, hypersexuality, compulsive shopping, binge‐eating, and related disorders such as hobbyism, punding, and abuse of dopaminergic medication.^1^ ICDs usually develop gradually over several months, due to a sensitization of the mesolimbic dopaminergic system.^2^, ^3^ O'Sullivan and colleagues (2010)^4^ were the first ones to describe a potentially related behavioral disorder they termed “reckless generosity” in three patients. Generosity is defined as “a willingness to give help or support more than is usual or expected.”^5^ Generosity is usually accompanied by a feeling of happiness and generally perceived positively by others. Here we report three cases of PD patients who developed reckless generosity under DRT, with devastating consequences on the patients' social lives and financial situation. To discuss possible underlying mechanisms of reckless generosity, we performed a narrative review of studies investigating the link between PD, dopamine and generous behavior.

## Case Series

All presented patients have a confirmed diagnosis of PD and were treated at the University Hospital of Bern between March 2019 and August 2020. ICDs and related behaviors were assessed by a neuropsychologist (D.A.) using the Ardouin Scale of Behavior in Parkinson's Disease (ASBPD).^6^ In this case series, the item “compulsive shopping” of the ASBPD was adapted to include questions on reckless generosity. It is noteworthy that we also observed milder forms of increased generosity in other PD patients under DRT. Informed consent was obtained from all patients.

### Case 1

A 49‐year old man, diagnosed with PD in 2014, self‐reported excessive generosity following an increase of ropinirole in the previous year. At the time of the consultation, he was on a regime of ropinirole 8 mg bid (total daily dose/TDD 16 mg) and L‐dopa 200 mg tds (TDD 600 mg), not suffering from any motor or non‐motor fluctuations. He had started to support three asylum seekers, helping them with administrative issues, letting one of them live in his house, and giving them money (equivalent to more than 15′000 CHF). He felt deep compassion for the three men and did not want to stop his generosity despite his limited financial resources. As a consequence, he borrowed money from his parents and overdrew his bank accounts. The year before, he had to leave his job after 33 years due to gross misconduct related to risky behavior, and his wife left him. In addition to excessive generosity, he also reported episodes of binge eating and a new‐found interest in creative hobbies. His libido had increased and he started seeking sexual encounters with men for the first time in his life. He had sexual intercourse with one of the asylum seekers occasionally, but stated that he never paid him for sexual favors and that the man did not seem to exchange sexual favors in the hope for financial support. No depressive symptoms or anxiety were reported. He met the criteria for hypomania, but not for mania according to DSM‐5. He consented to a reduction of ropinirole from 16 mg/day to 4 mg bid (TDD 8 mg) and an increase of L‐dopa to 700 mg TDD (administered in shorter intervals and distributed in four doses). Two months later he reported a decrease in generous behavior, libido and hypomania, experiencing no depressive symptoms or apathy.

### Case 2

A 47‐year old man, diagnosed with PD in 2017, developed several ICDs and reckless generosity under pramipexole 2.25 mg od, which had been reduced to 1.5 mg od before the neuropsychiatric assessment. He experienced no motor fluctuations. He worked as a taxi driver and had started to drive customers around for free, lending money to them occasionally, equivalent to several thousand Swiss francs at a time in some cases. He reported that he did not want to lend them money, but that he felt compassion for the people and that he simply could not decline their requests. He described that his character had changed from being shy and insecure to being self‐confident, jovial, and extroverted. He had started speculating on the stock market and had lost over 27′000 CHF within a couple of months. He had overdraws of over 3′000 CHF and had borrowed money from his brother. His libido had increased; he reported having more sexual fantasies, consuming pornography regularly, and starting to visit prostitutes. He stated that his generosity was not linked to his sexual encounters. At the time of consultation, he reported no anxious or depressive symptoms. He met the criteria for hypomania, but not for full‐blown mania according to DSM‐5. After pramipexole was substituted with L‐dopa 100 mg tds (TDD 300 mg), his motor state remained stable and his excessive generosity, speculation on the stock market, hypersexuality and hypomania gradually remitted, without showing any depressive symptoms or apathy.

### Case 3

A 56‐year old male, diagnosed with PD in 2011, was referred to our clinic to evaluate the possibility of deep brain stimulation (DBS). At the time of the first neuropsychiatric assessment, he was on a regimen of L‐dopa/carbidopa/entacapone 675/168.5/1000 mg distributed in five doses and safinamide 100 mg od, and experienced wearing off with prolonged OFF‐phases. He had developed reckless generosity under ropinirole 8 mg od, which persisted for another 5 months after ropinirole had been discontinued in 2018. Over 4 years, he had given an estimated 2.9 million CHF to his medical massage therapist, who had repeatedly besieged him to give her money. He stated that he felt very sorry for the massage therapist, as she told him that she would be financially ruined without his support. Later, when she started to pressure him by sending him multiple texts a day asking for money, he felt like he had to continue to support her despite not wanting to, to somehow get his money back in the end. He felt embarrassed about the situation and was hiding it from his wife. He never had an intimate relationship with the massage therapist and said that he was never interested in any sexual favors from her. His behavior felt inexplicable to himself, as he had built up a successful business in the past, never experiencing any financial trouble before. He had taken up several credits, borrowed money from friends, and had also started to access accounts from his company. At the time of the neuropsychiatric assessment, the debt enforcement office had announced that they would evict him and his wife from their apartment in the next few days. He was devastated, showing symptoms of anxiety and depression. However, he described that until he had stopped ropinirole, his mood had been elevated and that he had been a passionate racing driver. Despite a slightly increased libido in the relationship with his wife, he had not experienced any other ICDs. One year after the initial consultation, he reported that his financial situation had improved, although he still had some tax debts. He had taken legal action against the massage therapist and had managed to stay in his house. The reactive depressive symptoms and anxiety had improved, but not fully remitted, and he had gradually developed apathy. He received DBS 1.5 years after his initial consultation. His motor symptoms, depressive symptoms, anxiety, and apathy improved under DBS. On one occasion he lent 1000 CHF to an acquaintance, again after being pressured to do so. Other than that he reported no relapse.

## Literature Review

To further investigate the relationship between PD, respectively dopamine and generosity; we performed a Pubmed literature search according to PRISMA guidelines using the following advanced search strategy: (((“Parkinson Disease [MeSH Terms]”) OR (“Dopamine [MeSH Terms]”)) AND ((“Altruism [MeSH Terms]”) OR (“Genero* [Title/Abstract]”))). Additionally, references of the included articles were screened for eligible studies.

The following criteria had to be met for studies to be reviewed: studies had to be published in English between 1970 and August 2020 and had to examine the relationship between PD or dopamine and generosity or altruism. A total of 14 records were identified and assessed for eligibility, with six articles included in the review. An additional four articles were identified via screening of references of articles, with 10 studies finally included in the review.^4^, ^7^, ^8^, ^9^, ^10^, ^11^, ^12^, ^13^, ^14^, ^15^

Studies could be classified into the following categories: studies examining the relationship between PD and generosity; studies examining the relationship between dopaminergic medication and generosity; and studies examining the relationship between specific dopamine‐related genotypes and generosity.

### Parkinson's Disease and Generosity

One case series^4^ described reckless generosity in two male PD patients and one female PD patient. In all of the patients, reckless generosity was observed after treatment with pramipexole was initiated (two patients 6 mg/day and one patient 3 mg/day). All three patients were also taking L‐Dopa. One patient was additionally taking amantadine and another patient entacapone. In all three cases, excessive generosity developed along with other ICDs. While the female patient was generous only to family and friends, the two male patients were also generous towards strangers and casual acquaintances. Excessive generosity improved or remitted in all patients after pramipexole was discontinued or reduced.

One study^7^ tested PD patients with and without ICDs in a between‐group study design on and off dopaminergic medication in a trust task, and compared their performance with healthy controls. The trust task measured altruistic punishment, which is defined as punishing violators of social norms despite the personal cost. Overall, their results suggest that PD patients with ICDs are less sensitive to the aversive aspects of the lack of reciprocation in a trust task.

### Dopaminergic Medication and Generosity

Three randomized‐controlled studies^8^, ^9^, ^10^ examined the effect of dopaminergic medication on generous behavior in healthy persons, measured with variants of the Dictator Game; a task in which subjects decide to keep a certain amount of money for themselves or give some of it to people of varying degrees of social distance. One study^8^ examined the effect of 0.35 mg pramipexole and placebo on sharing behavior in women. Interestingly, after being administered pramipexole, women shared less with close loved ones, but more with socially distant people. In a similar study,^10^ either the dopamine‐antagonist amisulpride or placebo was administered to women and men before playing the Dictator Game. Their results suggest that women share less after taking a single dose of 400 mg amisulpride and that contrary, men share more after being administered a single dose of 400 mg amisulpride. Interestingly, those effects were only observed for close loved ones and not for socially distant people. Another study^9^ investigated the effect of 200 mg tolcapone and placebo on sharing behavior under different degrees of inequity. The results of this study suggest that even though the amount people were sharing did not change overall, there was a shift towards more egalitarian decisions after the administration of tolcapone.

### Dopamine‐Related Genotypes and Generosity

We included 5 studies examining generosity as a function of polymorphisms in dopamine receptors. Dopamine receptors can be grouped into two families according to their influence on the adenylyl‐cyclase activity. The activating D1 like‐family comprises D1‐ and D5‐receptors and the inhibitory D2 like‐family comprises D2‐, 3‐ and 4‐receptors. Dopamine agonists presently available for treatment nearly exclusively stimulate receptors from the D2 like‐family.^3^, ^16^ Most studies focused on the D4‐receptor (DRD4), which bears a variable number of tandem repeats in Exon III, with 2‐, 4‐ or 7‐repeats (R) being the most common polymorphisms. The 2‐ and 7‐R allele exhibit lesser responsiveness to dopamine than the 4‐R allele, and are associated with impulsivity, novelty‐seeking, and addiction.^17^, ^18^, ^19^

Two studies^11^, ^12^ found higher scores for generosity in healthy volunteers carrying the 4‐R allele or lacking the 7‐R allele respectively. In another study, carriers of the 7‐R allele showed larger differences in altruistic punishment between unfair and fair trials than non‐7‐R carriers when playing the Dictator Game.^13^ Furthermore, 7‐R allele carriers showed stronger reactions to unfair assignments. Two studies^14^, ^15^ found that secure attachment in children related to prosocial behavior only in carriers of the 7‐R allele, but not in non‐7‐R allele carriers.

In summary, these studies corroborate growing evidence that the 7‐R allele is a susceptibility allele in gene and environment interactions.

## Discussion

We report three cases of male PD patients who exhibited reckless generosity towards mere acquaintances under DRT treatment, leading to negative financial and social consequences. Interestingly, reckless generosity was accompanied by hypomania or an elevated mood and other ICDs in all three cases. However, in one of the cases with particularly devastating consequences, only very mild ICDs were reported. Even though hypersexuality was present in two of the cases and a slightly increased libido in one of the cases, the reckless generosity was not linked to sexual motives, but to compassion and an inability to say no to people. One patient compulsively invested money on the stock market, but despite their lavish generosity, none of the patients were pathological gamblers or compulsive shoppers. The behavior in one case resembled that of a gambling addict though, with the patient describing a feeling that he had to keep investing to make up for his losses. Overall, the described cases share many features with the reckless generosity first described in three patients by O'Sullivan and colleagues in 2010.^4^ Similar to the three cases described by them, reckless generosity in our cases was linked to intake of dopamine agonists, was accompanied by other ICDs, and subsided along with the other ICDs after dopamine agonists were reduced or discontinued. However, the patients described by O'Sullivan and colleagues^4^ had a longer disease duration and their reckless generosity was often aimed at family and friends and accompanied by pathological gambling or compulsive shopping. In our cases, reckless generosity seemed to appear regardless of disease duration and was aimed at mere acquaintances. The fact that we observed those severely disabling cases in a single movement disorders unit over a period of 18 months, suggests that this phenomenon is not rare and that the spectrum of known ICDs should be expanded to include reckless generosity. To enable screening for reckless generosity, an additional item or question should be included in the commonly used screening tools for ICDs (e.g. ASBPD, Questionnaire for Impulsive‐Compulsive Disorders in Parkinson's Disease, Minnesota Impulse Disorders Interview). One potential reason for altruistic behavior being an underreported side effect of DRT could be that it is usually perceived as a positive quality and that patients would not link this behavior to DRT. Furthermore, there is no definition of pathological generosity so far, and except for the description of excessive generosity in patients with bipolar disorder going through manic episodes, the phenomenon has not been described in psychiatry. Although studies using brain imaging and transcranial direct current stimulation in healthy individuals and case reports of patients with neurological conditions show that the mesocortical and mesolimbic dopaminergic systems are crucial for generous behavior,^20^, ^21^, ^22^, ^23^, ^24^, ^25^, ^26^, ^27^ studies examining the link between PD and generosity, respectively dopaminergic medication and generosity are still largely lacking. One available study suggests that PD patients with ICDs are less sensitive to the aversive aspects of the lack of reciprocation in social settings.^10^ This finding is in line with other studies showing that reward learning can be impaired in patients with ICDs.^28^ Two studies in healthy adults suggest that dopaminergic medication leads to reduced social discounting^8^ and more egalitarian behavior.^9^ However, another study suggests that the availability of dopamine inversely affects women and men, with men becoming more generous and women becoming less generous towards close loved ones after being administered a dopamine‐antagonist.^10^ Overall data from this study contradicted the two other studies suggesting that the availability of dopamine decreases sharing with close loved ones, but reduces social discounting in women. This might be explained by the fact that different types of dopaminergic medication were used in the studies. A general limitation of these studies is also that the effect of dopaminergic medication was not examined in the long‐term. This would be crucial, as studies show that ICDs and related behaviors develop gradually over time due to a sensitization of the mesolimbic dopaminergic system, which leads to an excessive response to dopaminergic stimulation.^29^, ^30^ In line with that, the improvement of ICDs, respectively the desensitization, usually also takes several months.^3^

Genetic studies show that polymorphisms in the DRD4 receptor have an impact on the strength of altruistic behavior^11^, ^12^ and on the degree to which generous behavior is susceptible to external stimuli.^14^, ^15^ Overall, these studies suggest that the mesolimbic (D3 receptor) and mesocortical (D4 receptor) dopaminergic system play an important role in the regulation of generous behavior. Whilst the mesolimbic bottom‐up processes such as reward‐seeking developed early on in evolution, the mesocortical, top‐down controlled processes such as reward learning are evolutionary younger.

Little is known about the relationship between cortical D4 receptors and ICDs, as most of the research focuses on the mesolimbic D3 receptor. Future research should investigate this relationship and examine different predictors and treatment options for reckless generosity in PD patients. In our case series, the discontinuation or reduction of dopamine agonist treatment led to a gradual improvement of reckless generosity and comorbid ICDs. If the driving mechanism for excessive altruism is a high affinity or sensitization of the D3/D4 receptors, not only a reduction of dopamine agonists but also clozapine, which is an atypical neuroleptic with an affinity for the D4 receptor,^31^ might be an effective treatment.

In summary, more research is needed to determine the prevalence and underlying mechanisms of reckless generosity in PD.

## Author Roles

1) Research Project: A. Conception, B. Organization, C. Execution; 2) Manuscript: A. Writing of the first draft, B. Review and critique.

D.A.: 1A, 1B, 1C, 2A

J.P.M.: 1A, 1B, 1C, 2A

I.D.: 2B

L.L.: 2B

J.M.: 2B

M.M.: 2B

K.S.: 2B

P.K.: 1A, 2A, 2B

## Disclosure

**Ethical Compliance Statement:** All procedures performed were following the ethical standards of the institutional and national research committee and with the 1964 Helsinki Declaration and its later amendment or comparable ethical standards. Written consent was obtained from all involved patients and the cases were sufficiently anonymized. We confirm that we have read the Journal's position on issues involved in ethical publication and affirm that this work is consistent with those guidelines.

**Funding Sources and Conflict of Interest:** No specific funding was received for this work. The authors declare that there are no conflicts of interest related to this work.

**Financial Disclosures for the Previous 12 Months:** D.A. has received a research grant from Parkinson Schweiz. J.P.M. has received honoraria as a speaker and travel reimbursements from Merz Pharma Switzerland; and travel reimbursements from Allergan Switzerland AG. I.D. has received a research grant from Boston Scientific, and traveling reimbursement from Zambon and Boston Scientific. P.K. has received research grants from Swiss National Science Foundation, ROGER DE SPOELBERCH Foundation, Bertarelli Foundation, Annemarie Opprecht Foundation, Parkinson Schweiz, Michael J Fox Foundation, Aleva Neurotherapeutics; and grants and personal fees (paid to institution) from Boston Scientific and BIAL. LL, J.M., M.M. and K.S. declare that there are no disclosures to report.

## References

[1] Weintraub D , Claassen DO . Impulse control and related disorders in Parkinson's disease. In: Chaudhuri KR , Titova N , eds. Nonmotor Parkinson's: The Hidden Face. International Review of Neurobiology. Vol 133. 1st ed. London, Oxford, Boston, New York, San Diego: Academic Press; 2017:679–717. 10.1016/bs.irn.2017.04.006. 28802938

[2] Voon V , Napier TC , Frank MJ , et al. Impulse control disorders and levodopa‐induced dyskinesias in Parkinson's disease: an update. Lancet Neurol 2017;16(3):238–250. 10.1016/S1474-4422(17)30004-2. 28229895

[3] Corvol JC , Artaud F , Cormier‐Dequaire F , et al. Longitudinal analysis of impulse control disorders in Parkinson disease. Neurology 2018;91(3):e189–e201. 10.1212/WNL.0000000000005816. 29925549 PMC6059034

[4] O'Sullivan SS , Evans AH , Quinn NP , Lawrence AD , Lees AJ . Reckless generosity in Parkinson's disease. Mov Disord 2010;25(2):221–223. 10.1002/mds.22687. 20077470

[5] *Cambridge Dictionary*. Cambridge University Press. https://dictionary.cambridge.org/de/worterbuch/englisch/generosity. Accessed December 11, 2020.

[6] Rieu I , Martinez‐Martin P , Pereira B , et al. International validation of a behavioral scale in Parkinson's disease without dementia. Mov Disord 2015;30(5):705–713. 10.1002/mds.26223. 25809278

[7] Djamshidian A , O'Sullivan SS , Doherty K , Lees AJ , Averbeck BB . Altruistic punishment in patients with Parkinson's disease with and without impulsive behaviour. Neuropsychologia 2011;49(1):103–107. 10.1016/j.neuropsychologia.2010.10.012.Altruistic. 20965203 PMC3005080

[8] Artigas SO , Liu L , Strang S , et al. Enhancement in dopamine reduces generous behaviour in women. PLoS One 2019;14(12):1–9. 10.1371/journal.pone.0226893. PMC6938376 31891605

[9] Sáez I , Zhu L , Set E , Kayser A , Hsu M . Dopamine modulates egalitarian behavior in humans. Curr Biol 2016;25(7):912–919. 10.1016/j.cub.2015.01.071.Dopamine. PMC4627633 25802148

[10] Soutschek A , Burke CJ , Raja Beharelle A , et al. The dopaminergic reward system underpins gender differences in social preferences. Nat Hum Behav 2017;1(11):819–827. 10.1038/s41562-017-0226-y. 31024122

[11] Bachner‐Melman R , Gritsenko I , Nemanov L , Zohar AH , Dina C , Ebstein RP . Dopaminergic polymorphisms associated with self‐report measures of human altruism: a fresh phenotype for the dopamine D4 receptor. Mol Psychiatry 2005;10(4):333–335. 10.1038/sj.mp.4001635. 15655563

[12] Anacker K , Enge S , Reif A , Lesch KP , Strobel A . Dopamine D4 receptor gene variation impacts self‐reported altruism. Mol Psychiatry 2013;18(4):402–403. 10.1038/mp.2012.49. 22565783

[13] Enge S , Mothes H , Fleischhauer M , Reif A , Strobel A . Genetic variation of dopamine and serotonin function modulates the feedback‐related negativity during altruistic punishment. Sci Rep 2017;7(1):1–12. 10.1038/s41598-017-02594-3. 28592831 PMC5462809

[14] Bakermans‐Kranenburg MJ , Van Ijzendoorn MH . Differential susceptibility to rearing environment depending on dopamine‐related genes: new evidence and a meta‐analysis. Dev Psychopathol 2011;23(1):39–52. 10.1017/S0954579410000635. 21262038

[15] Knafo A , Israel S , Ebstein RP . Heritability of children's prosocial behavior and differential susceptibility to parenting by variation in the dopamine receptor D4 gene. Dev Psychopathol 2011;23(1):53–67. 10.1017/S0954579410000647. 21262039

[16] Garcia‐Ruiz PJ , Martinez Castrillo JC , Alonso‐Canovas A , et al. Impulse control disorder in patients with Parkinson's disease under dopamine agonist therapy: a multicentre study. J Neurol Neurosurg Psychiatry 2014;85(8):841–845. 10.1136/jnnp-2013-306787. 24434037

[17] Eisenberg DTA , Campbell B , MacKillop J , Lum JK , Wilson DS . Season of birth and dopamine receptor gene associations with impulsivity, sensation seeking and reproductive behaviors. PLoS One 2007;2(11):e1216. 10.1371/journal.pone.0001216. 18030347 PMC2075470

[18] Ebstein RP , Novick O , Umansky R , et al. Dopamine D4 receptor (D4DR) exon III polymorphism associated with the human personality trait of novelty seeking. Nat Genet 1996;12(1):78–80. 10.1038/ng0196-78. 8528256

[19] Shao C , Li Y , Jiang K , et al. Dopamine D4 receptor polymorphism modulates cue‐elicited heroin craving in Chinese. Psychopharmacology (Berl) 2006;186(2):185–190. 10.1007/s00213-006-0375-6. 16703401

[20] Park SQ , Kahnt T , Dogan A , Strang S , Fehr E , Tobler PN . A neural link between generosity and happiness. Nat Commun 2017;8:1–10. 10.1038/ncomms15964. 28696410 PMC5508200

[21] Rilling JK , Sanfey AG , Aronson JA , Nystrom LE , Cohen JD . Opposing BOLD responses to reciprocated and unreciprocated altruism in putative reward pathways. Neuroreport 2004;15(16):2539–2543. 10.1097/00001756-200411150-00022. 15538191

[22] Liao C , Wu S , Luo Y , Guan Q , Cui F . Transcranial direct current stimulation of the medial prefrontal cortex modulates the propensity to help in costly helping behavior. Neurosci Lett 2018;674:54–59. 10.1016/j.neulet.2018.03.027. 29550374

[23] Zheng H , Huang D , Chen S , et al. Modulating the activity of ventromedial prefrontal cortex by anodal tDCS enhances the trustee's repayment through altruism. Front Psychol 2016;7(SEP):1–12. 10.3389/fpsyg.2016.01437. 27713721 PMC5031609

[24] Boes AD , Grafft AH , Joshi C , Chuang NA , Nopoulos P , Anderson SW . Behavioral effects of congenital ventromedial prefrontal cortex malformation. BMC Neurol 2011;11(1):151. 10.1186/1471-2377-11-151. 22136635 PMC3265436

[25] Moll J , De Oliveira‐Souza R , Basilio R , et al. Altruistic decisions following penetrating traumatic brain injury. Brain 2018;141(5):1558–1569. 10.1093/brain/awy064. 29590314 PMC7341482

[26] Sturm VE , Perry DC , Wood K , et al. Prosocial deficits in behavioral variant frontotemporal dementia relate to reward network atrophy. Brain Behav 2017;7(10):1–14. 10.1002/brb3.807. PMC5651391 29075567

[27] Ferreira‐Garcia R , Fontenelle LF , Moll J , de Oliveira‐Souza R . Pathological generosity: an atypical impulse control disorder after a left subcortical stroke. Neurocase 2014;20(5):496–500. 10.1080/13554794.2013.826681. 23962063

[28] Lee J‐Y , Jeon BS . Maladaptive reward‐learning and impulse control disorders in patients with Parkinson's disease: a clinical overview and pathophysiology update. J Mov Disord 2014;7(2):67–76. 10.14802/jmd.14010. 25360230 PMC4213534

[29] Evans AH , Pavese N , Lawrence AD , et al. Compulsive drug use linked to sensitized ventral striatal dopamine transmission. Ann Neurol 2006;59(5):852–858. 10.1002/ana.20822. 16557571

[30] Smith KM , Xie SX , Weintraub D . Incident impulsive control disorder symptoms and dopamine transporter imaging in Parkinson disease. J Neurol Neurosurg Psychiatry 2016;87(8):864–870. 10.1136/jnnp-2015-311827.Incident. 26534930 PMC4854795

[31] Seeman P , Corbett R , Van Tol HH . Atypical neuroleptics have low affinity for dopamine D2 receptors or are selective for D4 receptors. Neuropsychopharmacology 1997;16(2):93–110. 9015795 10.1016/S0893-133X(96)00187-X

